# Mental health screening in unaccompanied asylum-seeking children: screening tool selection and feasibility in the UK National Health Service

**DOI:** 10.1017/S1463423624000586

**Published:** 2025-04-10

**Authors:** Krsna Mohnani, Paula Seery, Hana Jayadel, Sophie Raghunanan, Alexandra M. Cardoso Pinto, Francesca Mathias, Dougal Hargreaves, Caroline Foster

**Affiliations:** 1 Department of Paediatric Infectious Diseases, Imperial College Healthcare NHS Trust, London, UK; 2 School of Medicine, Imperial College London, London, UK; 3 School of Public Health, Imperial College London, London, UK

**Keywords:** Asylum-seeker, mental health, refugee, screening, adolescents

## Abstract

There has been an increasing number of applications from unaccompanied asylum-seeking children (UASC) in the United Kingdom in recent years. It is well-known that this population is at high-risk of developing mental health disorders, which require early detection and intervention to facilitate successful integration. This paper describes the introduction of mental health screening for unaccompanied asylum-seeking children in a National Health Service (NHS) outpatient clinic in central London. This follows the results of a two-year retrospective analysis of the health needs of the population in our clinic, which identified a high incidence of disturbance to mood and sleep. We describe the selection process for a culturally appropriate and validated screening tool, piloting the Refugee Health Screener (RHS) tool with 20 UASC in clinic, and using preliminary findings to inform a more targeted referral to community Child and Adolescent Mental Health Services (CAMHS). We conclude that implementation of the RHS-13 is feasible for widespread mental health screening for UASC in an NHS setting, and provide suggestions for future research directions within this field.

## Introduction

Forced displacement presents a major challenge to our society with the global number of forcibly displaced people doubling over the past two decades, surpassing 100 million before the end of 2022 (United Nations High Commissioner for Refugees, 2022). Over 40% of forcibly displaced people are under the age of 18, many of whom are separated from their families during the journey, becoming what is termed an unaccompanied asylum-seeking child (UASC) (*see* Table 1 *–* Winchester, 2021; United Nations High Commissioner for Refugees, 2023). The United Kingdom has seen an increasing number of applications for asylum from UASC in recent years, receiving 4,382 applications in 2021 (GOV.UK, 2022a).

**Table 1.** Population definitions

Term	Definition
Forcibly displaced person	A person who is forced to move, within or across borders, due to armed conflict, persecution, terrorism, human rights violations and abuses, violence, the adverse effects of climate change, natural disasters, development projects or a combination of these factors.
Refugee	A person who has been forced to flee his or her country due to a well-founded fear of persecution for reasons of race, religion, nationality, political opinion or membership in a particular social group and is unable or unwilling to return.
Asylum seeker	A person who has applied for asylum and is awaiting a decision surrounding being granted refugee status.
Unaccompanied asylum-seeking child	A person under the age of 18 who is applying for asylum, has been separated from both parents and is not under the care of a legal guardian.

When an unaccompanied asylum-seeking child arrives in the UK, either spontaneously or via a refugee resettlement scheme, they are placed in the care of a local authority. Each local authority has the responsibility of meeting the child’s health, social care, housing, finance, and education needs. As with all Looked After Children (LAC), UASC undergo an Initial Health Assessment (IHA) within 20 working days of coming into care (National Institute for Health and Care Excellence, 2021).

At the start of the Covid-19 pandemic, due to ongoing concerns surrounding transmissible infections delaying housing placements with foster carers, the ‘Unity Clinic’ was set up for UASC cared for within the tri-borough of Westminster, Kensington and Chelsea and Hammersmith and Fulham. The clinic carried out infectious disease screening, baseline blood tests, as well as a general history and examination for UASC in a face-to-face manner despite COVID restrictions, in attempt to create a “one-stop shop”, a model of care which has been previously advocated for in the UK (Nezafat Maldonado et al., 2022). Analysis of the health needs of 155 UASC who attended our clinic between November 2019 and March 2022 found that over one third had an infection requiring recall, most commonly latent tuberculosis, strongyloides and hepatitis B. Additionally over half disclosed traumatic incidents prior to arrival in the UK, with 46.8% reporting ongoing disturbance to their mood and/or sleep (Cardoso Pinto et al., 2022).

It is the recommendation of the National Institute of Clinical Excellence that a validated, brief screening tool should be used to assess for post-traumatic stress disorder in refugees and asylum seekers, whilst the Royal College of Paediatrics and Child Health stress the importance of supporting young persons in accessing mental health care (National Institute for Health and Care Excellence, 2018; RCPCH Health Policy Team, 2018). Furthermore, a comprehensive ten-year review called for urgent early recognition, intervention, and access to mental health services for asylum seeking, refugee and undocumented children in Europe (Kadir et al., 2019). It has been recommended that initial assessments could be better adapted to recognise mental health difficulties in the context of migration (Dubs et al., 2022).

Previously, mental health screening within the Unity Clinic and IHAs included unstructured questions around mood, sleep and appetite as part of an adolescent HEEADSSS assessment, a process which may introduce cultural bias (Snowden, 2003; Goldenring & Rosen, 2004). It was subsequently concluded it was important to introduce a standardised tool validated for migrant youth, in order to reliably assess the mental health needs of adolescents attending the Unity Clinic.

The aim of this project was therefore to:

1. Review existing mental health screening tools described in the literature.
2. Investigate feasibility of an appropriate tool in this setting and population.
3. Use preliminary findings to inform service improvement and a more targeted referral to Child and Adolescent Mental Health Services (CAMHS).

## Methods

### Literature review of tools

There is no international consensus around gold-standard screening for mental health disorders in forced migrant populations. However, several tools have been described within the literature. As per Poole et al., screening tools should be self-reported or administered by trained Health and Social Care staff, responsive to change, with proven reliability and validity and a minimal response burden (Poole et al., 2020). The above criteria were agreed in consultation with our team psychologist, with the addition of ensuring to screen for both depression and PTSD, given that these are the most widespread disorders reported within this group and yet many tools fail to do this (Hocking et al., 2018). For practical uses within the Unity Clinic, the ideal tool also needed to be time efficient and applicable to a population inclusive of 14- to 18-year-olds, the age range of more than 95% of UASC attending the service (Cardoso Pinto et al., 2022).

To identify the appropriate screening tool, a literature search was conducted on PubMed, using the following search terms and combination operators:

Refugee OR “asylum seeker” OR migrant OR “displaced person” OR “Refugees”[Mesh]

**AND**

Trauma OR “health status” OR “mental health” OR depression OR anxiety OR “Post-traumatic stress disorder” OR PTSD OR “Mental Health”[Mesh]

**AND**

Measur* OR screen* OR tool OR assess* OR instrument OR questionnaire OR survey OR psychometric OR “Surveys and Questionnaires”[Mesh]

Results were filtered for studies which were reviews, systematic reviews, meta-analyses, and books and documents, published in English in the last 20 years. Clinical trials, case studies and other article types were excluded from our search.

This yielded a total of 531 studies. Titles were reviewed, and if unclear, the study abstracts were consulted, yielding 21 studies describing mental health screening tools in migrant populations. From this list, three were excluded due to reviewing tools specifically about grief (Killikelly et al., 2018), tools in Arabic only (Zeinoun et al., 2021), or digital tools (Liem et al., 2021), leading to a total of 18 studies. 18 full texts were read and 13 of these were deemed relevant, as in Table 2.

**Table 2.** Thirteen studies included in review with title, authorship, and date of publication

Author	Study Title
Donnelly and Leavey (2021)	Screening tools for mental disorders amongst female refugees: a systematic review
Magwood et al. (2022)	Mental health screening approaches for resettling refugees and asylum seekers: a scoping review
Gagliardi et al. (2021)	Health-related quality of life of refugees: a systematic review of studies using the WHOQOL-Bref instrument in general and clinical refugee populations in the community setting
Kameg (2019)	Management of mental health conditions in refugee youth: An overview for the psychiatric-mental health nurse practitioner
Turner et al. (2019)	Clinical tools working at home with immigrants and refugees
Horlings and Hein (2018)	Psychiatric screening and interventions for minor refugees in Europe: an overview of approaches and tools
Dowling et al. (2017)	Measuring self-rated health status among resettled adult refugee populations to inform practice and policy – a scoping review
Gadeberg et al. (2017)	Assessing trauma and mental health in refugee children and youth: a systematic review of validated screening and measurement tools
Sigvardsdotter et al. (2016)	Refugee trauma measurement: a review of existing checklists
Gagnon and Tuck (2004)	A systematic review of questionnaires measuring the health of resettling refugee women
Gulbrandsen et al. (2004)	Identification of need for psychiatric help among asylum seekers
Hollifield et al. (2002)	Measuring trauma and health status in refugees: a critical review
Uysal-Bozkir et al. (2013)	Insufficient cross-cultural adaptations and psychometric properties for many translated Health Assessment Scales: a systematic review

Overall, 295 screening tools were discussed in these 13 papers, or 198 tools when duplicates and alternative versions of the same tool were removed. Tools were subsequently included if they assessed both trauma and mood, with an age range of 14–18 years, were available for use, and were developed for/validated in refugee and asylum-seeking populations.

The measurement focuses and age ranges of each tool were either obtained from tool descriptions in systematic review papers, or from publicly available information. If these metrics were unavailable, the age ranges of participants in study samples were used instead.

**Figure 1.**
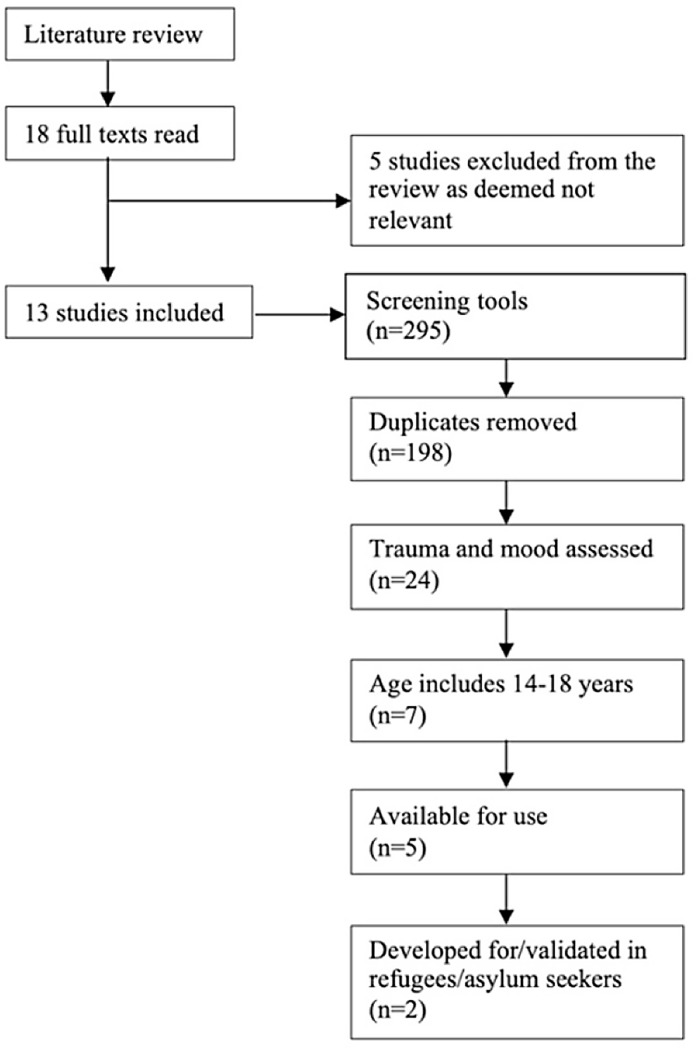
Flowchart outlining process in selecting screening tool.

The flowchart is mapped out in Fig. 1, with all papers and tools listed in *Supplementary File 1*.

The two tools remaining following the application of our criteria were the Digital Communication Assistance Tool (DCAT) and the Refugee Health Screener (RHS).

The **Digital Communication Assistance Tool (DCAT)**, described by Müller et al. in 2020 (Müller et al., 2020), is an app-based tool for use in the waiting room, consisting of 1800 different items in its query algorithm that explores common health complaints for refugees in 19 different languages and dialects. Whilst it does address mental health symptoms, it covers predominantly physical health symptoms and was therefore deemed to be outside the scope of our search.

The **Refugee Health Screener (RHS)** is a highly sensitive, efficient, and culturally responsive screening tool for common mental disorders, available as RHS-15, and the later developed, RHS-13. It has been validated for refugee populations in multiple groups and been translated into 15 languages using a “rigorous, iterative back-and-forth participatory consensus process with refugees from each language group” (Hollifield et al., 2013, 2016; Polcher & Calloway, 2016; Kaltenbach et al., 2017; Bjärtå et al., 2018; Fellmeth et al., 2018). Only about 10% of UASC attending the Unity Clinic speak English (Cardoso Pinto et al., 2022). The tool can be self-administered or administered by healthcare professionals (HCP), interpreters or others involved in patient care and does not require a trained mental health professional (Refugee Health Technical Assistance Center, 2012). It has been proven useful for large-scale use, is used in several sites worldwide and is suggested by the Centers for Disease Control and Prevention in their published guidance for newly arrived refugees (Hollifield et al., 2016; Stingl et al., 2017; Centers for Disease Control and Prevention, 2022).

The **RHS-15** consists of 15 items with excellent internal consistency (α  =  0.92), made up of 13 symptom items rated on a 5-point Likert scale, 1 coping item and one distress thermometer, measuring distress on a visual analogue scale ranging 0 to 10. It was developed by Pathways to Wellness in 2011, using the comprehensive New Mexico Refugee Symptom Checklist-121 and selecting items that were thought to be most predictive of anxiety, depression, and PTSD (Pathways to Wellness, 2011). Further items were added based on clinical experience and empirical data and the Hopkins Symptom Check List-25 and the PTSD Scale-Self Report for DSM-5 were used as diagnostic proxies (DPs) given their validity in predicting depression and anxiety, and PTSD respectively. Of note, it does not ask any details of the traumatization to avoid additional distress or potential harm. Its full development and validation are described by Hollifield et al. (Hollifield et al., 2013).

A ‘positive’ case on the RHS-15, supported by previous post-hoc testing, is indicated by a score of ≥12 (or an item average of 0.88) in the first fourteen questions, or a score of ≥5 in the distress thermometer. The **RHS-13** is a shortened version involving only the first thirteen questions, for use if time is a key consideration. It has a cut-off for a positive case at ≥11. Analysing the predictive validity of both, the sensitivity/specificity ratio for an RHS-15 case is 0.98/0.77, whilst this is 0.96/0.86 for an RHS-13 case (Hollifield et al., 2016). The authors, however, also provide sensitivity and specificity data with various cut-off scores for both versions, to allow organisations to consider variations in scoring based on local resources.

Having satisfied the above criteria, the RHS-15 was selected as our primary tool of use for the clinic, with the option of moving to RHS-13, depending on initial pilot timings.

### Piloting the refugee health screener tool

The RHS-15 was piloted with consent with every UASC who attended the Unity Clinic and/or follow up paediatric infectious disease appointments at St. Mary’s Hospital in Paddington, London, between September and November 2022. Participants were selected due to their attendance during this time frame, with no further exclusion criteria.

This population included 20 young people from a diverse range of countries, and varying time spent in the UK. In cases where only the month of arrival was known and not the exact date, the 15^th^ day of the month was used as a proxy. In general, the majority of participants were of male gender and originated from the horn of Africa, with ages varying between 14 and 17 years. The questionnaire was carried out towards the end of each clinic appointment, either with support from an interpreter, or without if the UASC was literate or was able to understand English. Results were recorded in the Trust’s electronic patient record system, CernerEPR. Full demographics and results of the pilot sample are included in Table 3.

**Table 3.** Characteristics of pilot sample

Gender	Male	19
	Female	1
Age	14	1
	15	2
	16	12
	17	5
Nationality	Sudan	5
	Eritrea	5
	Ethiopia	4
	Syria	2
	Algeria	1
	Sierra Leone	1
	Afghanistan	1
	Albania	1
Time since arrival in UK	Less than 1 month	3
	≥ 1 month and < 6 months	10
	≥6 months and < 12 months	5
	≥ 12 months < 18 months	2
RHS-15 results (n = 7) in *Q1–14*	0–10	4
	11–17	1
	18–24	2
	25–57	0
	With median score of 2 out of 10 on *Q15* (distress thermometer*)*	
RHS-13 results (n = 13)	0–10	4
	11–17	2
	18–24	2
	25–52	5

Based on the first seven pilots, the RHS-15 took between 6 and 12 min to administer. Question 15, the distress thermometer or visual analogue, proved to be time-consuming and challenging to be understood, as has been the case in others’ experiences, and may reduce specificity by generating false positives (Hollifield et al., 2016; Salt et al., 2017; Stingl et al., 2017; Baird et al., 2020).

The visual analogue scale proved particularly challenging to explain when booked face-to-face interpreters were unable to attend and telephone interpreting services used instead. The authors state that if efficiency is a key consideration, leaving out the last two questions may be acceptable from a metric perspective. Due to time constraints in clinic, and the similar psychometric properties described for RHS-13, a decision was made to move to RHS-13 for the seventh interview onwards.

The mean time of administration of RHS-13 was 5 min and 32 s (range of 2–10 min). In general, screening times were longer when conducted with a telephone interpreter, and shorter if the UASC could speak English and/or was literate in their native language. The full anonymised dataset including language spoken is available in *Supplementary File 2*.

### Informing service improvement

Given both the limited healthcare resources and the increasing number of forcibly displaced populations, introducing cut-off scores is useful in guiding prioritization of patients when carrying out screening. The RHS authors too recognise that a site may opt for a higher cut-off score, if preference is for greater specificity given fewer services available (Hollifield et al., 2013).

In their paper investigating RHS-13, Bjärtå et al. suggest three cut-offs evaluated against the symptom scales of the Patient Health Questionnaire (PHQ-9) for depression, Generalised Anxiety Disorder Assessment (GAD-7) for anxiety and Primary Care PTSD Screen for DSM-5 (PC-PTSD-5) for PTSD (Bjärtå et al., 2018) – mild/subclinical, moderate and severe. ROC analysis for the moderate cut-off resulted in a high AUC of 0.915 (*P* < 0.001, 95% CI 0.885–0.945), with the greatest specificity with at least 70% sensitivity at a **score of 18.** Similarly, analysis of the severe cut-off showed an AUC of 0.855 (*P* < 0.001, 95% CI 0.823–0.887), with highest specificity and again at least 70% sensitivity at a **score of 25**.

Screening utility of the RHS-13 cut-offs by the proxy (i.e., a positive result on at least one of the proxies) with corresponding sensitivity/specificity ratios are as listed in Table 4.

**Table 4.** RHS-13 cut-off scores alongside screening proxy indices and sensitivity/specificity ratios, as per Bjärtå et al. (2018)

RHS-13	Screening proxy index	Sensitivity/specificity ratio
Score of 11	≥5 on PHQ-9, ≥5 on GAD-7 and ≥2 on PC-PTSD-5	0.85/0.94
Score of 18	≥10 on PHQ-9, ≥8 on GAD-7 and ≥3 on PC-PTSD-5	0.72/0.93
Score of 25	≥15 on PHQ-9, ≥15 on GAD-7 and ≥4 on PC-PTSD-5	0.71/0.82

In the literature, ≥10 on the PHQ-9 is suggested as the point at which it to stop watchful waiting, and initiate a “treatment plan”, with a sensitivity of 88% and a specificity of 88% for major depression (Kroenke et al., 2001). A score ≥8 on the GAD-7 is described as a “reasonable cut-point for identifying probable cases of generalized anxiety disorder”, with sensitivity of 76% and specificity of 92% (Kroenke et al., 2007; Plummer et al., 2016). A cut-off of three on the PC-PTSD-5 has been found to be optimally sensitive to suggest probable PTSD, with a sensitivity of 95% and a specificity of 85%, and has been used in other UK-based studies (Prins et al., 2016; Kar et al., 2021).

With this knowledge, it was decided to opt for ≥10 on PHQ-9, ≥8 on GAD-7 and ≥3 on PC-PTSD-5, or correspondingly an RHS-13 **score of 18 as our primary cut-off** for screening. Bjärtå et al. describe this point as “clinically significant” (Bjärtå et al., 2018).

From Hollifield et al.’s data, a cut-off score of 18 has a good sensitivity with a range 0.687–0.820, and excellent specificity with a range 0.935 to 0.972, when compared to the diagnostic proxies for PTSD, depression, and anxiety. This cut-off is slightly less sensitive than the authors’ original cut-off of 11 but naturally more specific (sensitivity range 0.82–0.96 and specificity range 0.862 – 0.906 for cut-off score 11) (Hollifield et al., 2016).

Our **secondary cut-off for screening is 25** and is described as “in acute need of advanced care” by Bjärtå et al. who found that most individuals with severe symptoms had mental health problems at diagnostic levels after attending further assessment. Leiler et al. build on this finding, emphasising the need to carry out a suicide risk assessment in all, but most especially for individuals scoring ≥25 should resources be scarce (Leiler et al., 2019).

The screening pathway below was subsequently produced in consultation with the department’s Looked-After-Children, Child and Adolescent Mental Health Services and Infectious Diseases team:

- If a UASC scores 18–24, safety netting advice is provided and their carer and social worker is asked to monitor their mental health, after obtaining consent from the young person and their General Practitioner (GP) informed by letter.
- If a UASC scores 25 or higher, their case is discussed with the lead clinician on-site. The LAC medical team urgently refer to CAMHS, or if at a residence out-of-borough, urgently inform the UASC’s GP so they may be referred to their local CAMHS service. Safety netting advice is given, and the young person is assessed for signs of an acute crisis.

Any young person presenting with acute mental health needs is referred for urgent assessment irrespective of RHS-13 screening score via the established Accident and Emergency pathway.

**Figure 2.**
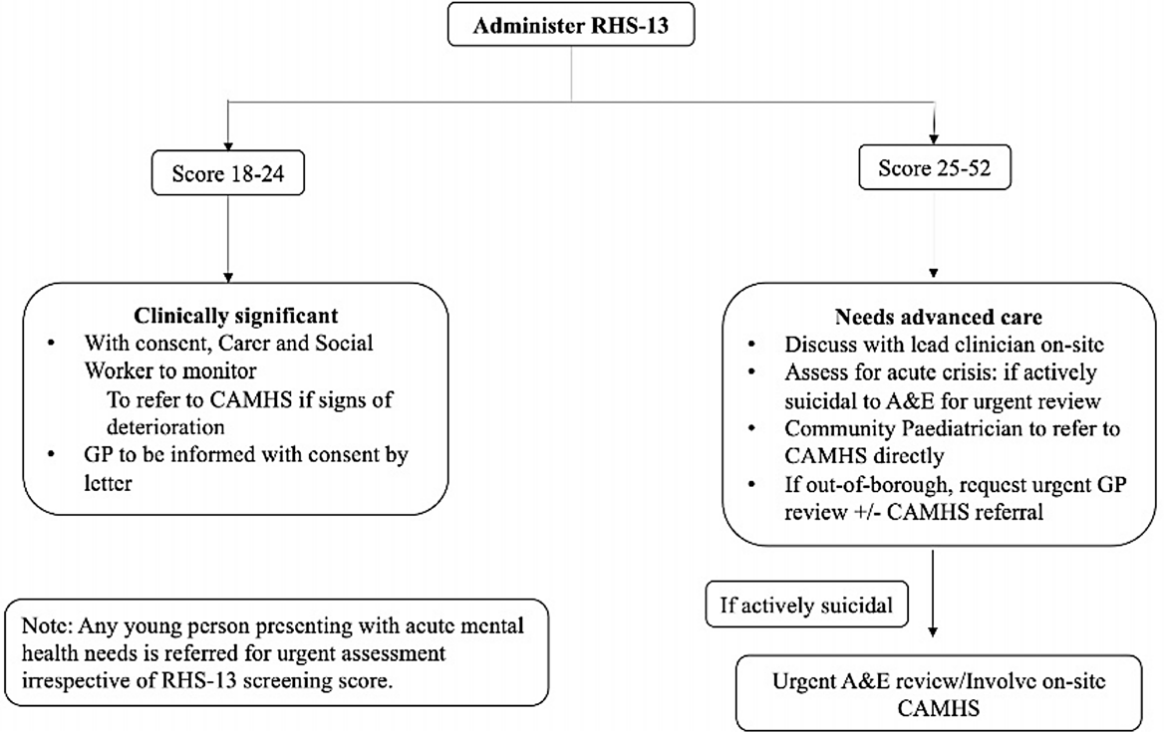
Referral pathway following administration of RHS-13.

The referral pathway was outlined and subsequently introduced to the team, as it appears in Fig. 2 below.

## Discussion

The mental health of asylum-seekers and refugees is a pressing topic within policy, academia, and clinical practice. The Unity Clinic data is not a standalone finding. UASC data from Kent showed that 41% of UASC experienced mental health symptoms, and this number rises to 77% in Camden, London (Coyle & Bennett, 2016; Armitage et al., 2021). One study in Oxford found that more than a quarter of refugee children have significant psychological disturbance, about three times the national average (Fazel, 2003). In Europe, a systematic review of 47 studies across 14 countries showed the upper ranges of prevalence in asylum-seeking adolescents are 52.7% for post-traumatic stress disorder (PTSD), 32.8% for depression, 31.6% for anxiety and 35% for emotional/behavioural symptoms (Sommer et al., 2018). This may be due to traumatic experiences pre-migration, challenges during migration, as well as issues with integration, discrimination, poverty and limited social support on resettlement, particularly whilst dealing with complex legal processes and frequent relocation, with post-migration stressors aggravating or worsening mental health status (Nielsen et al., 2008; Goosen et al., 2013; Rousseau & Frounfelker, 2018; Müller et al., 2019; Giannopoulou et al., 2022). It should be noted that unaccompanied children tend to be at greater risk of mental health conditions than their accompanied asylum-seeking peers (Pinto Wiese & Burhorst, 2007; Hodes et al., 2008; Bean et al., 2007; Derluyn et al., 2009; Stotz et al., 2015; Norredam et al., 2018). Yet, a study conducted in one of our own local boroughs found that most UASC are not in contact with mental health services (Sanchez-Cao et al., 2012).

It is unsurprising then that the World Health Organisation calls for collective action to protect and promote the health needs of these vulnerable populations (United Nations High Commissioner for Refugees, 2022). Screening of mental health, in particular, should be viewed as a “high priority” in receiving communities, in order to prevent further decompensation and facilitate triage to appropriate services (Hocking et al., 2018; Kadir et al., 2019; Eiset et al., 2020; Daniel-Calveras et al., 2022).

Moreover, it is argued that early detection, coupled with evidence-based treatment, is not only compassionate but also cost-effective, resulting in benefits for both the individual and society at large (Kaltenbach et al., 2017; Bjärtå et al., 2018). This is what encouraged the team to introduce a screening tool into our population, and, in particular, one that was going to able to sensitively pick up mental health symptoms in our UASC in a brief and culturally responsive way.

NHS England and each local authority hold the shared responsibility of ensuring UASC, as all looked-after children, are registered with a GP – which is encouraged to be undertaken as soon as they arrive in the UK (NHS Kent and Medway Looked After Children’s Team, 2019; GOV.UK, 2022b). Within Primary Care, service providers have found “difficulties in finding a common ground to talk”, and have called for “clear referral and care pathways within the mental health service” for this population (Misra et al., 2006). It is hoped that the RHS and its accompanying scoring system will provide a universal recognition of distress for UASC, facilitating communication across all specialist, GP and social services. In addition, after the Initial Health Assessment, subsequent mental health concerns from carers are managed through the GP with view to CAMHS referral – hence it could also be worth considering expansion of the RHS to Primary Care too.

Learning from colleagues across the UK, a national review recognised the Tavistock and Portman NHS Foundation Trust as the only specialist mental health service for UASC, that screens for mental health problems using the Strengths and Difficulties Questionnaire (SDQ) when the young person first enters the care system in Haringey, and thereafter annually (Tavistock and Portman NHS Trust, 2020; Dubs et al., 2022). In the North East of England, Harkensee et al. describe screening UASC via psychosocial risk and protective factors and subsequently introducing SDQ into their clinic (Harkensee & Andrew, 2021). Finally, in Camden, London, the team mention recognising the limitations of screening using the SDQ and state that their CAMHS service has recently shifted to the RHS-15 (Armitage et al., 2021). Worldwide, the Refugee Health Screener has been used in a variety of studies, ranging from administration in clinical settings to communal accommodations, refugee centres and even reception facilities, although the original authors of the tool feel that healthcare settings are ideal as stigma is likely to be less (Refugee Health Technical Assistance Center, 2012). A positive screen is usually described as being referred for a diagnostic interview and evaluate the need for specific treatment (Stingl et al., 2017; Shedrawy et al., 2019). One study described introducing the *Pathways to Wellness* intervention for those that screened positive, created by the authors of the RHS-15 and made up of eight facilitated 90-minute sessions, but this did not seem to show any statistically significant benefit post-intervention (Salt et al., 2017). Gerber et al. describes how participants who screen positive were given a printed list of local providers in English depending on specific participant needs (Gerber et al., 2017). Whilst some teams seem to delegate follow-up to the caseworker or Youth Welfare Office (Salt et al., 2017; Felsman et al., 2019; Hanewald et al., 2020), other studies describe referral to an on-site psychologist or a HCP first (Miletic et al., 2006; Polcher & Calloway, 2016; Kaltenbach et al., 2017). Ballard-King et al. describe administration of RHS-15 in local clinics in Kentucky, where a Mental Health Coordinator receives referrals from positive screens in clinic, as well as referrals from community staff with concerns (Ballard-Kang et al., 2017). Similarly, our pathway incorporates both options where the community social worker is asked to monitor the UASC’s mental health and can refer to CAMHS if needed, whilst a healthcare professional can also refer to CAMHS directly, a two-pronged approach that depends on the outcome of RHS-13 screening.

When considering the NHS in particular, time and resources are often the main limiting factors to service improvement. For this reason, pilot interviews were timed, and the shorter yet equally sensitive version of the RHS tool was selected for long-term use. UASC attending the Unity Clinic also have full Initial Health Assessments as for a Looked After Child and infectious disease screening with blood and urine sampling, balancing the need for a one stop shop approach and not overburdening young people attending. Generally, administration of the RHS-13 took just over 5 min, in line with the authors’ experience of 5–15 min, although it has taken longer in some studies (Lutheran Community Services Northwest et al., 2011; Hollifield et al., 2013; Nationalities Service Center, 2014; Kaltenbach et al., 2017). Secondly, “categorizing” by level of risk has been found to reduce the response burden, prompting the introduction of cut-offs in our referral pathway (Magwood et al., 2022). Direction of resources to those with the greatest need is particularly important given the significant pressure CAMHS is operating under, which needs to be addressed with urgency (Dubs et al., 2022). In our small sample, 38.5% of young people scored above 25 in the RHS-13 and were offered referral to CAMHS. Finally, it should be noted that since the carer, social worker and GP also play an active role in monitoring the UASC’s mental health, all adults involved in the care of the UASC should receive mental health awareness training, a concept that has been previously advocated for by Lord Dubs and others (McDonald et al., 2021; Dubs et al., 2022).

## Strengths and limitations

The greatest strength of this pilot is that, to our knowledge, it is the first study to describe the pilot use of the Refugee Health Screener in a setting within the United Kingdom’s National Health Service. It is hoped that forming part of the conversation on mental health screening, as well as transparency on integrated pathways, may encourage others to hold similar discussion in their own Trusts. The absence of mental health expertise on-site, as well as the fact that the RHS-13 can be administered by both HCP and non-HCPs, may also make our work more generalisable to other institutions in comparable situations.

Our work, however, should also be considered in the context of important limitations. Firstly, the majority of screening was conducted with the help of telephone or face-to-face interpreters. It must be noted that there was variation in approach and timing of each translator, and interpreters too are subject to misinformation about mental health (Miletic et al., 2006). Secondly, whilst attempts were made to conduct the screening interviews in the absence of social workers and foster carers, UASC themselves may not be forthcoming to healthcare professionals due to stigma associated with mental health in immigrant communities, mistrust towards adults and fear of compromising their asylum application. When support or referral is offered, it may be declined for similar reasons, as is often the case (Savin et al., 2005; Polcher & Calloway, 2016; Demazure et al., 2017). In terms of timing of assessment, a “honeymoon” period has been described, which is a period of euphoria experienced on arrival which lasts about 1–3 months – screening has sometimes been done intentionally outside of this period (Polcher & Calloway, 2016). In our pilot study, screening was done for all those attending the Unity Clinic and/or follow-up appointments for convenience, which resulted in varying lengths of time from date of arrival in the UK across interviews, mostly between 1 and 6 months from arrival. Whilst timely recognition and treatment is important, the severity levels of RHS-13 do appear sensitive to change and may be more accurate later in the resettlement process (Bjärtå et al., 2018; Baird et al., 2020; Hollifield et al., 2021). Discussions around the best time for screening and room for repeat screening is ongoing, and should be revisited over time (Lutheran Community Services Northwest et al., 2011; Magwood et al., 2022). Finally, since the aim of the pilot study was purely to understand feasibility, we used a small sample of 20 UASC and do not explore acceptability or effectiveness of the RHS-13 tool nor appropriateness of the defined cut-offs in this sample itself – which we acknowledge remain key to this discussion.

## Conclusions and future directions

In conclusion, culturally validated, time-sensitive mental health screening tools exist and are readily available for routine use with unaccompanied asylum-seeking adolescents. Such tools, like the RHS-13, can be feasibly implemented in an NHS outpatient setting, together with appropriate pathways to address identified needs.

A major challenge within the field is the lack of record-level data for unaccompanied children seeking asylum and subsequently limited awareness of the complexity of their needs, something that the Children’s Commissioner of England has recently been advocating for (de Souza, 2023). Whilst this service improvement aimed to only introduce mental health screening, plans are in place to evaluate long-term physical and mental health follow-up for each of these children, including assessment of access and delivery which is lacking (Pollard & Howard, 2021). Secondly, as mentioned above, it would be useful to investigate overall effectiveness of screening and appropriateness of defined cut-offs, as well as if any correlation exists between RHS-13 scores and certain demographics. This warrants a systematic study with larger, probabilistic sampling in order to draw precise and accurate conclusions on these parameters. Furthermore, is not uncommon for UASC to be eventually moved outside of the local authority they are initially placed under, in line with equitable distribution policies of the National Transfer Scheme (GOV.UK, 2022c). For those that were referred to CAMHS, future efforts should therefore focus on continuation of service (and preventing loss to follow-up) once moved outside of London, as well as during transition from the LAC care system after the age of 18. There may also be value in exploring the role of third sector charities in plugging the gaps in UASC health and social care – a sector many believe the full potential of which has not been fully realised in the NHS (Bull et al., 2014). Finally, given the rise of patient and public involvement (PPI), it would be helpful to understand both the acceptability of RHS-13 screening and the value of services offered to UASC, from their perspective. The aim is to involve our patients into the design of their own future service improvement and ultimately use their views in a way that informs best practice.

## Supporting information

Mohnani et al. supplementary material 1 Mohnani et al. supplementary material

Mohnani et al. supplementary material 2 Mohnani et al. supplementary material

## Supplementary material

For supplementary material/s referred to in this article, please visit https://doi.org/10.1017/S1463423624000586
